# MRI, CT and FDG-PET/CT findings of Wolffian tumor: four-case series

**DOI:** 10.1007/s11604-021-01145-1

**Published:** 2021-06-05

**Authors:** Hisataka Ito, Takashi Koyama, Yuichiro Kanie, Kozue Morioka, Moto Nakaya, Akihito Mitsumori, Sakiko Kageyama, Masashi Kusakabe, Kaori Kuriyama

**Affiliations:** 1 Department of Diagnostic Radiology, Kurashiki Central Hospital, 1-1-1 Miwa, Kurashiki, Okayama 710-8602 Japan; 2 Department of Radiology, Japanese Red Cross Society Himeji Hospital, 1-12-1 Shimoteno, Himeji, Hyogo Japan; 3 Department of Radiology, Yamagata Prefectural Central Hospital, Aoyagi, Yamagata-shi, 1800, Yamagata, Japan; 4 Department of Radiology, NTT Medical Center Tokyo, 5-9-22 Higashi-Gotanda, Shinagawa-ku, Tokyo, Japan; 5 Department of Diagnostic Radiology, Tohoku University Graduate School of Medicine, 2-1 Seiryo-machi, Aoba-ku, Sendai, Miyagi Japan

**Keywords:** Wolffian tumor, Female adnexal tumor of probable Wolffian origin, MRI, CT, PET/CT

## Abstract

**Purpose:**

The purpose of this study is to describe the characteristic MRI, CT, and ^18^F-fluorodeoxyglucose-positron emission tomography/computed tomography (FDG-PET/CT) findings of Wolffian tumor.

**Methods:**

We reviewed preoperative images in four surgical cases of Wolffian tumor. MRI was available for review in all cases with additional diffusion-weighted images (DWI) in three, and contrast-enhanced images in two. CT was available in three. FDG-PET/CT was obtained in two.

**Results:**

Two patients were asymptomatic, while the other two presented with acute abdomen. On MRI, all tumors were well-defined masses of increased signals on T2WI. Three tumors were solid, whereas the other was solid and cystic. The normal ipsilateral ovary was identified in two patients of reproductive ages, but not in two postmenopausal patients. Tumors in two patients presented with acute abdomen were complicated by hemorrhage. All three tumors evaluated on DWI showed high intensities. Contrast-enhanced images of MRI and CT showed homogeneous enhancement as the same degree as the myometrium. On CT, one tumor contained punctate calcifications. FDG-PET/CT showed moderate FDG accumulation.

**Conclusion:**

Wolffian tumors may be typically solid extraovarian tumors occasionally associated with cysts and calcifications. Although they are benign, they mimic malignancy due to high intensities on DWI and increased FDG accumulation.

## Introduction

The Wolffian tumor is an extremely rare and a newly designated tumor in the 2014 World Health Organization classification of tumors of female reproductive organs. This tumor was initially described as a female adnexal tumor of probable Wolffian origin (FATWO) in 1973 by Kariminejad and Scully [1]. One of the reasons for the assumption of Wolffian origin is that this tumor commonly occurs in the broad ligament, in which Wolffian remnants are abundant. The other reason for this assumption is the lack of histologic resemblance to other tumors of Müllerian origin.

Patients are usually asymptomatic or presented with non-specific abdominal symptoms when the tumor is large enough [2]. The tumor is equally seen in both reproductive and menopausal women. The mean age at diagnosis is reported to be 50 years ranges from 15 to 83 years [3]. There are no specific biomarkers or hormonal production by this tumor.

Histologically, Wolffian tumor is characterized by the proliferation of small to medium-sized neoplastic cells that demonstrate solid, tubular patterns in a highly cellular manner. Occasionally, sieve-like cyst formation may be encountered. Nuclear atypia and mitoses are rare; however, the presence of necrosis, capsular invasion, a high rate of mitoses, cellular pleomorphism are considered to be the properties of malignant potential [4]. Pathologically, Wolffian tumor should be carefully differentiated from other neoplasms, particularly with endometrioid carcinoma and sex cord-stromal tumors such as Sertoli-Leydig cell tumor and granulosa cell tumor (GCT). Because of this variety of histologic patterns, the role of intraoperative pathologic consultation for establishing diagnosis is very limited.

Wolffian tumor is basically regarded as benign, although malignant behavior with distant metastasis and recurrence are seen in approximately 20% of cases [5]. Owing to the rareness of this tumor, the imaging findings of this tumor have been limited in sporadic case reports. In the previous case reports, Wolffian tumor is described as a well-defined solid or solid and cystic mass in the adnexa [6–9]. The reported magnetic resonance imaging (MRI) signals of the solid component are iso-intense to the skeletal muscles on T1-weighted images (T1WI), and intermediate intensities on T2-weighted images (T2WI), homogeneous enhancement on contrast-enhanced images, and high intensity on diffusion-weighted images (DWI) with restricted apparent diffusion coefficient (ADC) titer [6–9]. In one case report, the rim of hypointensities at T2WI has been reported as a characteristic imaging finding [9]. At computed tomography (CT), the tumor shows predominantly soft-tissue attenuation as same as the skeletal muscle, and contains curvilinear and punctate calcification [8].

Hereby, we present MRI, CT, and ^18^F-fluorodeoxyglucose-positron emission tomography/computed tomography (FDG-PET/CT) findings in four cases of Wolffian tumor, aiming to describe the characteristics and spectrum of the imaging findings.

## Materials and methods

### Patient profiles

Our case series consists of four surgical cases of Wolffian tumor from four institutes, those were presented in the 33rd annual meeting of the Japanese Society of Abdominal Radiology. The institutional review board approved this retrospective study, and no individual patient consent was required. The age of the four patients were 27, 39, 60, and 66 years old, with an average of 48 years old. Preoperative MR examinations were undergone in all four patients, and the mean interval between the MRI and the surgery was 37 days (range:0–92 days). Three patients also underwent CT, and the mean interval between the CT and the surgery was 66 days (range:5–112 days). FDG-PET/CT was obtained in two patients, and the interval between the FDG-PET/CT and the surgery was 22 and 40 days. The radiologists from each institute inquired about the clinical information of the patients.

### Imaging protocols

MRI examinations were performed using 1.5-T or 3.0-T systems. At least, axial T1WI (TR:400–679 ms; TE:8.3–13.0 ms; slice thickness:3.5–7 mm; slice interval:5–8.4 mm) and both axial and sagittal T2WI (TR:3300–6528 ms; TE:84–108 ms; slice thickness:3.5–7 mm; slice interval:3.0–7.8 mm) were obtained in all cases. Additionally, DWI (TR:3000–7200 ms; TE:72–97 ms; slice thickness:3.5–7.0 mm; slice interval:3.0–8.4 mm; *b* value:800–1000 s/mm^2^) were scanned in three patients. ADC value was measured in two of the cases, though it was not available in the other case. Contrast-enhanced MR images were obtained in two patients utilizing fat-suppressed T1WI (TR:400–759 ms; TE:8.3–13.0 ms; slice thickness:3.5–7 mm; slice interval:3–8.4 mm).

Three patients also underwent CT. CT examinations were performed on multidetector (64 or 80) row CT scanners with a reconstructed slice thickness of 5 mm. Contrast-enhanced CT was performed in one patient, and images were obtained at 120 s after intravenous administration of the contrast agent.

FDG-PET/CT was obtained in two patients. Combined PET/CT images were obtained one hour after administration of FDG of 228 or 250 MBq. Unenhanced CT images were acquired with a slice thickness of 1or 3.75 mm.

### Image analysis

Radiologists from each institute evaluated radiologic images. Finally, two radiologists of one institute who have experiences in abdominal radiology of 2 and 26 years, respectively, re-evaluated the radiologic images of all cases. The discrepancies in the image evaluation were resolved by conference manner.

Initially, the location and size of the tumors were evaluated on MRI of all patients. The following characteristics were assessed: predominant signal intensities of the tumor on both T1WI and T2WI in comparison to the skeletal muscles, the presence of cystic components of high intensities, rim of hypointensities on T2WI, hemorrhage of hyperintensities on T1WI, contrast enhancement on contrast-enhanced images. On DWI, in addition to the assessment of the signal intensities, the ADC titer was measured in the solid components of the tumor.

CT images were evaluated regarding the presence of calcification, cystic components and the attenuation of solid components compared to the skeletal muscles.

Concerning FDG-PET/CT, FDG uptake in the tumor was evaluated both visually and quantitatively by measuring maximum standardized uptake value (SUV)s.

## Results

### Clinical findings

**Fig. 1:**
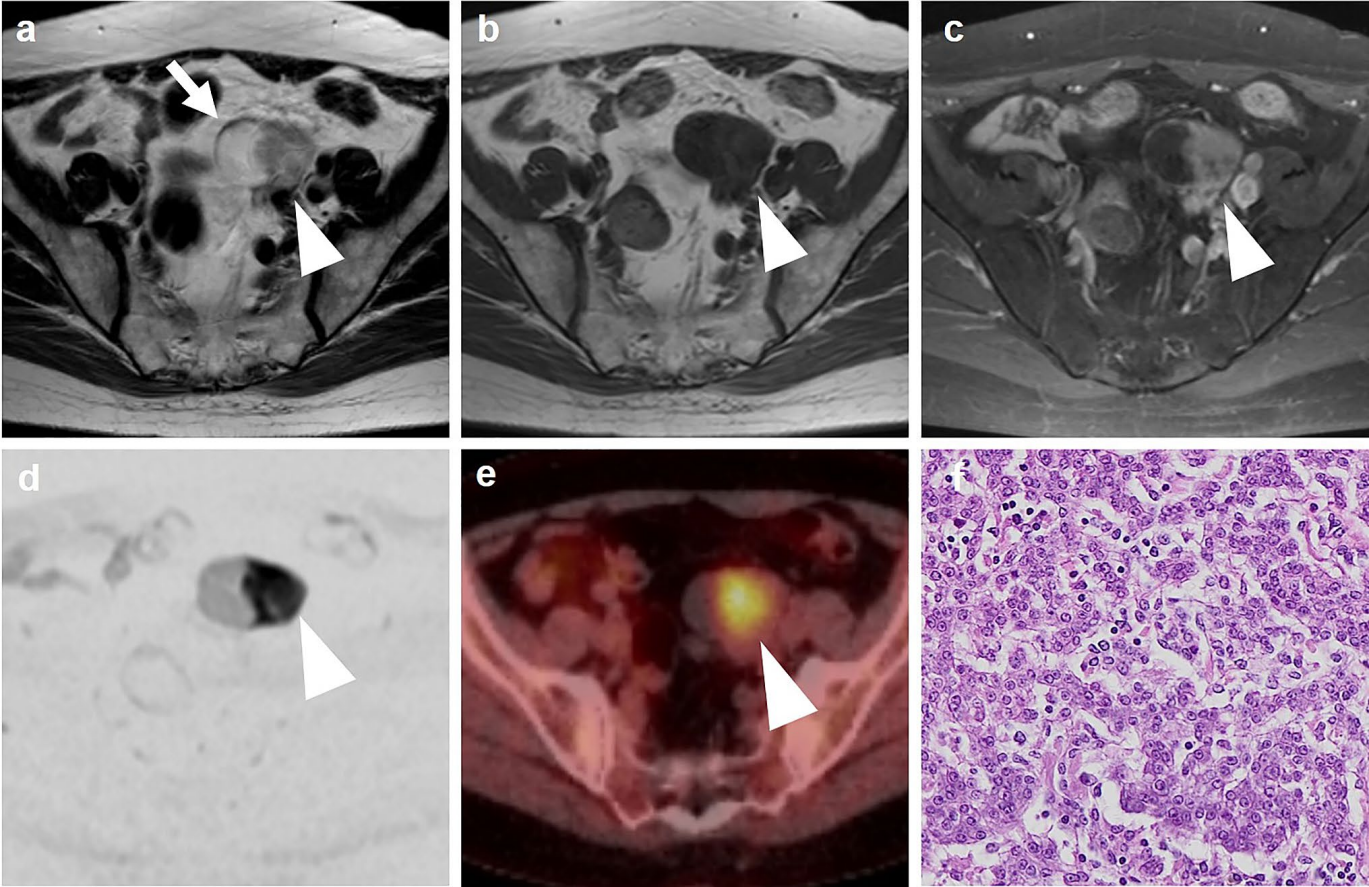
A 60-year-old asymptomatic woman who has a history of follicular lymphoma. **a** Axial T2-weighted MR image demonstrates a well-defined tumor (arrowhead) of intermediate intensities located along the left pelvic wall, containing a cystic area (arrow) of high intensities. **b** T1-weighted image shows the tumor (arrowhead) of iso-intensities. **c** Fat-saturated post-contrast T1-weighted image shows homogeneous enhancement (arrowhead) as same degree as the myometrium. **d** Diffusion-weighted image at *b* value of 800 with inverted black and white gray scale shows increased signals in the tumor (arrowhead). **e** The fused PET/CT image shows FDG accumulation in the solid component (arrowhead), measuring SUV max of 4.2. **f** Microphotograph of the specimen reveals the proliferation of epithelioid cells arranged in trabecular pattern. Tumor cells contain a moderate amount of eosinophilic cytoplasm and round or ovoid nuclei

**Fig.2:**
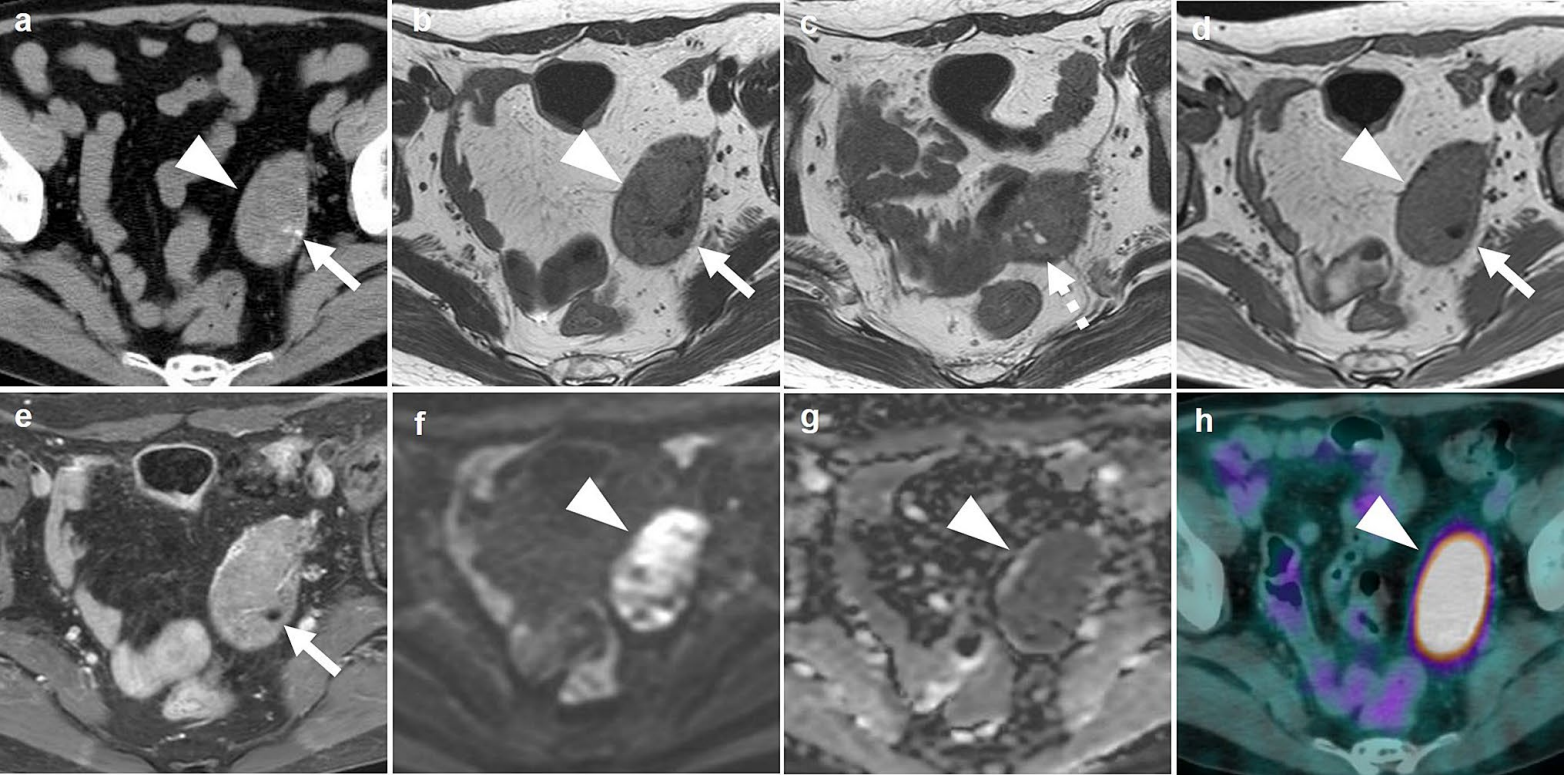
A 66-year-old asymptomatic woman who has diabetes mellitus. **a** Axial unenhanced CT shows a well-defined tumor (arrowhead) of predominantly soft-tissue attenuation, containing small foci of calcification (arrow). **b** Axial T2 weighted MR image demonstrates an entirely solid tumor (arrowhead) of intermediate intensities along the left pelvic wall, containing an area of hypointense dot corresponding to the calcification on CT (arrow). **c** Another axial T2-weighted image at caudal level shows cystic components of hyperintensities (dashed arrow). **d** T1-weighted image shows the tumor of iso-intensities (arrowhead) compared with an area of hypointense dot corresponding to the calcification (arrow). **e** Fat saturated contrast-enhanced T1-weighted image shows homogeneous enhancement in the solid portions except for the focus of calcification (arrow). **f** Diffusion-weighted image at b-value of 1000 shows heterogeneous high intensities within the tumor (arrowhead). **g** ADC map image shows decreased apparent diffusion coefficient (ADC) value (0.57 × 10^–3^ mm^2^/s) in the tumor (arrowhead). **h** The fused PET/CT image shows homogeneous FDG accumulation, measuring SUV max of 8.2 (arrowhead).

**Fig. 3:**
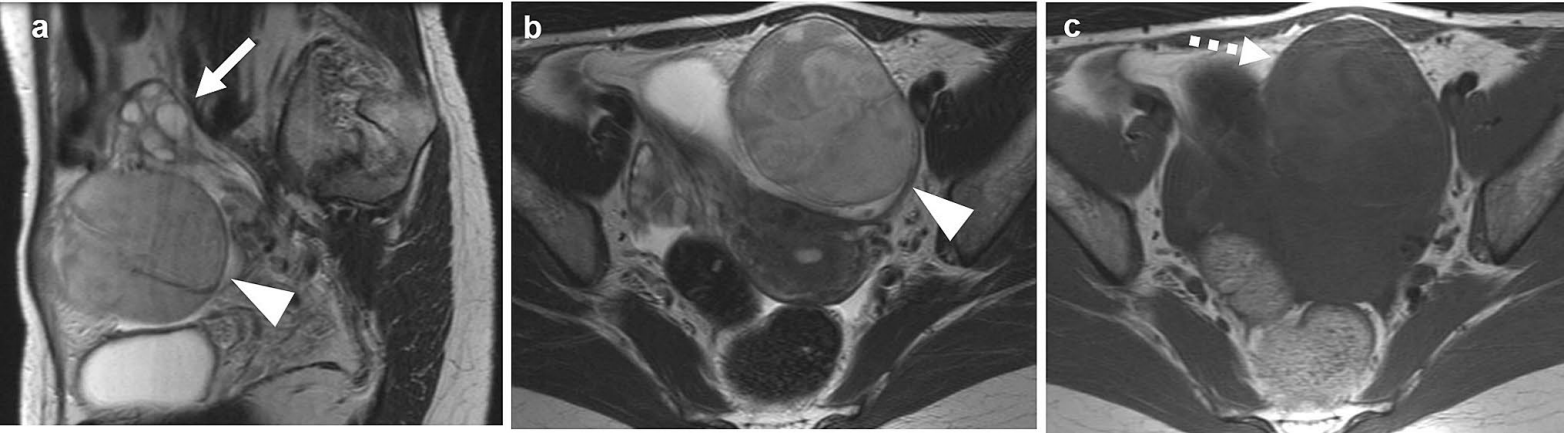
A 39-year-old woman presented with left lower abdominal pain of acute onset. **a** Sagittal T2-weighted image shows a tumor (arrowhead) located caudal to the left ovary (arrow). The tumor is enveloped by a thin layer of hypointensity, which is considered to be a fibrous capsule. **b** Axial T2-weighted MR image shows a well-defined solid tumor (arrowhead) of intermediate intensities ventral to the uterus. **c** Axial T1-weighted image shows areas of increased signals within the tumor (dashed arrow), suggestive of hemorrhage. Although torsion of the tumor was suspected by this finding, it was not apparent at surgery

**Fig. 4:**
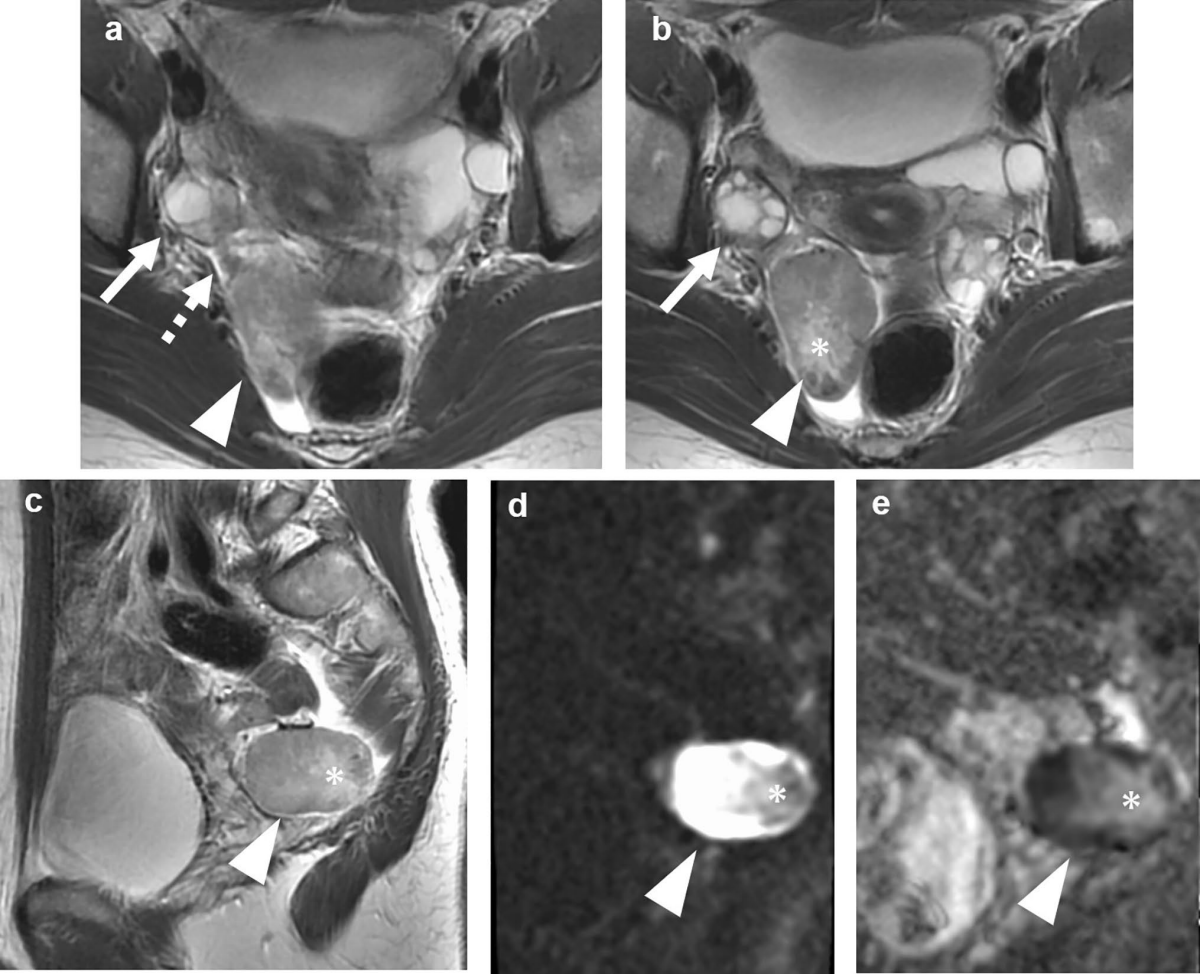
A 27-year-old woman presented with right lower abdominal pain of acute onset. **a** Axial T2-weighted MR image shows a well-defined solid tumor (arrowhead) posterior to both the uterus and right ovary (arrow), associated with twisted structure between the tumor and right ovary (dashed arrow). **b** Another axial T2-weighted image of caudal level shows a central area of increased intensities (asterisk), which is suspected of necrosis caused by torsion. The rest of the tumor is intermediate intensities. **c** Sagittal T2-weighted image shows tumor of intermediate intensities (arrowhead) containing areas of increased intensities suggestive of necrosis (asterisk). **d** Diffusion-weighted image at b-value of 800 shows the tumor of high intensities (arrowhead) except for areas of decreased signals in necrosis (asterisk). **e** ADC map image shows decreased ADC value (1.0 × 10^–3^ mm^2^/s) in the solid component except for areas suspected of necrosis of increased ADC value (2.3 × 10^–3^ mm^2^/s) (asterisk)

Two patients were asymptomatic (Figs. 1, 2), whereas the other two patients presented with acute onset of lower abdominal pain (Figs. 3, 4). In the former asymptomatic patients, the tumors were incidentally found during routine examinations for diabetes mellitus and malignant lymphoma, respectively. Their laboratory data were unremarkable except for blood glucose and hemoglobin A1c titer in the patient with diabetes. In the latter symptomatic patients, laboratory data were unremarkably except for elevated white blood cell count in one patient. Tumor markers, including CA 19–9 and CA 125, measured in two patients were within normal range.

### MR Imaging findings

Imaging findings of four cases are summarized in Table 1. MRI demonstrated well-defined adnexal masses in all patients, ranging from 50 to 70 mm with averaged size of 56 mm. Three tumors were solid, whereas the other was solid and cystic. They were located in the left adnexa in three cases (Figs. 1–3), and in the right adnexa in the other case (Fig. 4). The ipsilateral normal ovaries were clearly identified beside the tumors in two patients of reproductive age (Figs. 3, 4). In one patient, the tumor was identified posterior to both the uterus and right ovary (Fig. 4). In the other, the tumor was ventral to the uterus and caudal to the left ovary (Fig. 3). In the remaining two patients, in whom the tumors were located along the left pelvic wall, the shrunk ipsilateral ovary was difficult to identify because of the postmenopausal age of these patients (Figs. 1, 2). Peritoneal implants and enlarged lymph nodes were not recognized in any patients.

**Table1:** Imaging findings of four cases

Figure	Laterality	Size(mm)	T1WI	T2WI	DWI	ADC(mm^2^/s)	CT	Contrast enhancement	Cyst	Calcification	Hemorrhage	SUV max
1	Left	50	Iso	Intermediate	Hyper	NA	Iso	Homogeneous	Presence	Absence	Absence	4.2
2	Left	54	Iso	Intermediate	Hyper	0.57 × 10^–3^	Iso	Homogeneous	Presence	Presence	Absence	8.2
3	Left	70	Iso to slightly Hyper	Intermediate	NA	NA	NA	NA	Absence	Absence	Presence	NA
4	Right	50	Iso	Intermediate to hyper	Hyper	1.0 × 10^–3^	Iso	NA	Absence	Absence	Presence	NA

*Iso* iso-intensities, *Intermediate* intermediate intensities, *Hyper* hyper intensities, *Hypo* hypo intensities, *NA* not available, *CT* attenuation or MRI intensities is in comparison with skeletal muscles

In two asymptomatic patients, the tumors were iso-intense to skeletal muscles on T1WI, and intermediate intensity on T2WI. Both tumors included cystic components of hyperintensities on T2WI (Figs. 1, 2). Fat saturated contrast-enhanced T1WI, which were available only in these two patients, showed homogeneous enhancement (Figs. 1, 2).

In one patient with acute abdominal pain, T2WI demonstrated a twisted structure between the normal ovary and the tumor, suggestive of torsion and the tumor origin outside the ovary (Fig. 4). The central area of the tumor was intermediate intensity on T2WI, which was suspected of necrosis caused by torsion. The rest of the tumor showed a similar signal pattern to the previous cases. In these three patients above, DWI were available for review and demonstrated high intensities with decreased ADC values on the solid components of the tumor. In one tumor complicated by torsion, the central area showed decreased signals on DWI and increased ADC value, which was corresponded to the area of increased intensities on T2WI (Fig. 4). The ADC values of the solid component were 0.57 × 10^–3^ and 1.0 × 10^–3^ mm^2^/s respectively.

In the other symptomatic patient, the tumor contained massive areas of slightly increased intensities on both T1WI and T2WI suggestive of intra-tumoral hemorrhage (Fig. 3). However, no findings suggestive of torsion were identified. The tumor was enveloped by a thin layer of hypointensity on T2WI, which was considered to be a fibrous capsule (Fig. 3). In the other patients, there were no apparent rim of hypointensities on T2WI.

### CT findings

Unenhanced CT in the three patients showed adnexal masses of soft-tissue attenuation as same as the skeletal muscles. Two tumors in asymptomatic patients contained cystic areas of water attenuation, as MRI described above.

### FDG-PET/CT findings

FDG-PET/CT available for review in two asymptomatic patients showed moderate FDG accumulation in the tumors with SUV max of 4.2 and 8.2, respectively (Figs. 1, 2).

### Surgical findings

Three patients underwent unilateral salpingo-oophorectomy, while one asymptomatic patient underwent bilateral salpingo-oophorectomy, and hysterectomy or lymphadenectomy was not performed in any cases. All tumors were surgically treated as benign since the tumors were well-defined, and peritoneal implants and enlarged lymph nodes were not recognized in any patients on preoperative imaging findings. Surgically confirmed locations of the tumors were the left broad ligament (Fig.1), left ovarian hilum (Fig. 2), left mesosalpinx (Fig. 3), and right mesosalpinx (Fig. 4). In all cases, the normal ovaries were surgically confirmed.

In one patient with clinically suspected torsion, the tumor located in the right mesosalpinx was twisted 720° involving the right fallopian tube (Fig. 4). In the other patient with suspected intratumoral hemorrhage, any findings of torsion were not apparent during surgery (Fig. 3).

### Pathology

In all four cases, pathologic findings of the tumors were the same and characterized by the proliferation of epithelioid cells arranged in anastomosing tubular patterns or trabecular patterns (Fig. 1f). All tumors were diagnosed as benign and lacked a high mitotic rate or cytologic atypia. One tumor complicated with torsion exhibited necrosis and hemorrhagic infarction. In the case of suspected intratumoral hemorrhage on MRI, pathology also demonstrated infarcted tumor cells and edematous stroma as well as intratumoral hemorrhage. These findings may suggest preceding torsion. In all cases, tumors do not have intraluminal or intracytoplasmic mucin, nuclear grooves, and Call-Exner bodies.

## Discussion

Our case series show that Wolffian tumors were typically demonstrated as predominantly solid or cystic-solid adnexal tumors apart from the normal ovaries. In the English literatures, MRI findings of Wolffian tumor had been described in five cases in four case reports [6–9]. In these reports, the signal intensities on T1WI were iso-intense to the skeletal muscles, and those on T2WI were intermediate intensities in all cases. Three of five tumors included cystic components of hyperintensities on T2WI [6–8]. These signal patterns of MRI were similar to those observed in asymptomatic patients in our series.

In our case series, any tumor did not show the apparent rim of hypointensities at T2WI, which has been reported as a characteristic finding in one case report [9]. A thin layer of hypointensity noted in one of our case (Fig. 3) was fibrous capsule, and its appearance was quite different from that in the case report.

Our case series also demonstrates that Wolffian tumor showed increased signals on DWI with restricted ADC in the solid components. The same signal patterns were also observed in three patients in two case reports [8, 9]. The restricted diffusion in this tumor is considered to represent one of the pathologic characteristics, that is, high cellularity of this tumor.

There have not been any reports describing the imaging findings of FDG-PET/CT. Usually, adnexal tumor of increased FDG uptake is considered as malignancy in the postmenopausal woman. It is reported that cut-off value of SUV max of 2.55 is best to separate benign from borderline/malignant ovarian tumors on FDG-PET/CT [10]. Another study reports that the SUV max of 4.0 may be the best cut-off titer for differentiating malignant from benign or borderline ovarian tumors [11]. In our two cases evaluated by FDG-PET/CT, both tumors showed moderately uptake of FDG more than 4. These two cases suggest that the increased FDG uptake does not mean a malignancy, at least in cases of Wolffian tumor.

In only one case report describing the unenhanced CT findings, the tumor showed predominantly soft-tissue attenuation as same as the skeletal muscles and contained curvilinear and punctate calcification [8]. Brenner tumor also commonly contains calcification. However, the pattern of calcification is usually dense or amorphous, which may be different from that of Wolffian tumor.

The extraovarian location, namely the broad ligament origin, is an important clue to suspect Wolffian tumor for the adnexal tumor since the signal patterns on MRI and CT attenuation of the tumor themselves are non-specific and may not allow differentiation from other tumors. The normal ovaries of reproductive age could be identified at MRI in two patients in our case series. In the literature, the extraovarian locations were apparent in all tumors in reproductive women [6, 8, 9]. When the normal ovaries could be detected outside the tumor, the differential diagnoses of solid adnexal tumors include exophytic ovarian tumors, tubal carcinoma, tumors originating from the broad ligament such as leiomyoma or gastrointestinal stromal tumor (GIST) occurring in the mesenterium. Tubal carcinoma is typically associated with dilated fallopian tube, peritoneal implantations, and increased titer of CA125. On the other hand, myoma of the broad ligament typically exhibits markedly low signals on both T2WI and DWI. However, it may be difficult to distinguish from cellular myoma which demonstrates intermediate intensities on T2WI and hyperintensities on DWI. Subserosal myomas may also present as adnexal tumors, but they can usually be differentiated by its continuity with the uterus. GIST of mesenteric origin can occur in the pelvis. The tumor may contain hemorrhage, necrosis, and cystic change, particularly in large tumors [12]. The continuity to the intestine or mesenteric vessels will be the key finding to suspect GIST. Ovarian fibroma is the most common subtype of sex cord-stromal tumors. The tumor can be demonstrated as an exophytic ovarian tumor with preservation of the normal shape of the ipsilateral ovary, especially in reproductive women [13]. This tumor typically exhibits low intensity on T2WI reflecting spindle shape fibroblastic cells and abundant collagen and hyalinized tissue. However, it may be difficult to distinguish Wolffian tumor from degenerated fibroma demonstrating increased signals on T2WI or cyst formation.

If the normal ovaries cannot be detected on MRI because of postmenopausal age of the patients, the differential diagnoses will include solid ovarian tumors composed of high cellularity, such as GCT, Sertoli-Leydig cell tumor, and Krukenberg tumor. FDG-PET/CT findings may be useful in differentiation from GCT because most primary GCT demonstrates low FDG activity. Although other sex cord-stromal tumors such as fibroma, thecoma, and Brenner tumor can present as solid tumors, these tumors typically exhibit low intensity on T2WI reflecting fibrous histology.

Two patients in our series presented with acute lower abdominal pain. The tumor in one patient was complicated by the torsion. The intratumoral hemorrhage seen in the other patient may also result from proceeding torsion of the tumor. In the literature, one case of torsion of Wolffian tumor has been reported [6]. Wolffian tumor should be considered in the differential diagnosis of twisted adnexal tumors, especially when the ovaries were normally recognized.

The limitation of our case series is a limited number of cases. This is probably due to the extreme rareness of the tumor, and the reported imaging findings are limited to case reports in the English literature. However, we considered the imaging findings of Wolffian tumor in our series, and those in the literature were similar, and thus, they represent characteristics of this rare tumor.

## Conclusion

Wolffian tumor is typically demonstrated as a predominantly solid adnexal tumor with increased signals on both T2WI and DWI apart from the normal ovaries. This tumor tends to show moderate uptake of FDG even though it is benign. Wolffian tumor should be considered in the differential diagnosis for solid adnexal tumor in reproductive women in whom the tumor is apart from the normal ovary. However, in postmenopausal women in whom MRI fails to show extra ovarian location of the tumor, differentiation from other solid ovarian tumors will be difficult. In Wolffian tumor, the increased FDG uptake more than 4 does not mean malignancy.
